# Is population subdivision different from speciation? From phylogeography to species delimitation

**DOI:** 10.1002/ece3.6524

**Published:** 2020-06-28

**Authors:** Jen‐Pan Huang

**Affiliations:** ^1^ Biodiversity Research Center Academia Sinica Taipei Taiwan

**Keywords:** phylogeography, population subdivision, speciation, speciation continuum, species delimitation

## Abstract

Species‐level diversity and the underlying mechanisms that lead to the formation of new species, that is, speciation, have often been confounded with intraspecific diversity and population subdivision. The delineation between intraspecific and interspecific divergence processes has received much less attention than species delimitation. The ramifications of confounding speciation and population subdivision are that the term speciation has been used to describe many different biological divergence processes, rendering the results, or inferences, between studies incomparable. Phylogeographic studies have advanced our understanding of how spatial variation in the pattern of biodiversity can begin, become structured, and persist through time. Studies of species delimitation have further provided statistical and model‐based approaches to determine the phylogeographic entities that merit species status. However, without a proper understanding and delineation between the processes that generate and maintain intraspecific and interspecific diversity in a study system, the delimitation of species may still not be biologically and evolutionarily relevant. I argue that variation in the continuity of the divergence process among biological systems could be a key factor leading to the enduring contention in delineating divergence patterns, or species delimitation, meriting future comparative studies to help us better understand the nature of biological species.

## Graphical abstract

Is population subdivision a speciation process? The answer to the question impacts how we delineate intraspecific and interspecific diversities and may differ across biological systems. Understanding the continuity of divergence processes in different biological systems can be a key to answer the question.

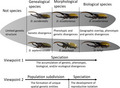

## 1 INTRODUCTION

Is population subdivision a speciation process? The difficulty in delineating, or distinguishing, biological divergence processes can be best exemplified by comparing the terminology used in the examples of parallel adaptation in three‐spine sticklebacks (McKinnon & Rundle, 2002) and crater‐lake cichlids in Central America (Elmer et al., 2014). Both systems developed after the last glacial age, when many new freshwater niches became available for colonization. Parallel evolution in the sticklebacks repeatedly led to limnetic and benthic ecological forms in the same lake across many different lakes (Jones et al., 2012). Parallel evolution in the cichlids, however, led to multiple sympatric pairs of limnetic and benthic “species” in different lakes (Elmer et al., 2014). Did biological, ecological, or genomic differences between the stickleback and cichlid fishes lead to more divergence in the cichlids, prompting new species? Or were cichlid taxonomists more prompted to describe new species? Note that the stickleback system has long been regarded as a model system for “sympatric speciation” in nature, although the different ecological forms of the sticklebacks have rarely been taxonomically treated as different species (McKinnon & Rundle, 2002). That is, the mechanisms in the stickleback system that only structure intraspecific genetic variation, or population subdivision, are considered speciation.

**FIGURE 1:**
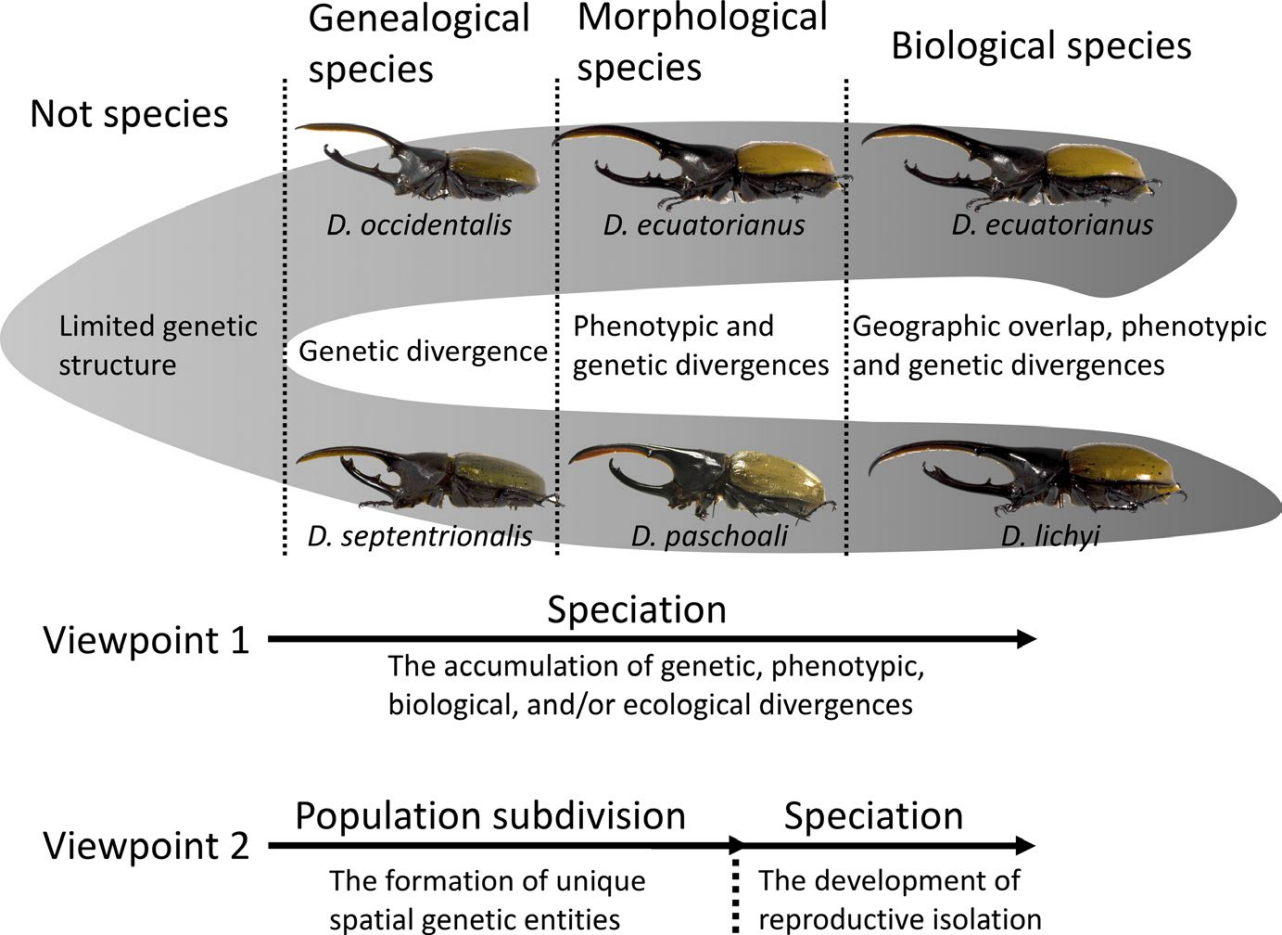
An example of a continuous divergence process, different species designations, and two viewpoints of delineating the divergence process from the Hercules beetles. The difference is whether processes that generate intraspecific divergence is speciation. *Dynastes occidentalis* (Chocó‐Darién ecoregion) and *Dynastes septentrionalis* (Central American cloud forest) are allopatric sister taxa that show significant genetic divergence, but they are morphologically similar to each other. *Dynastes ecuatorianus* (Northwestern, i.e., Colombian, Ecuadorian, and Peruvian, Amazonian rainforest) and *Dynastes paschoali* (Atlantic Forest) are allopatric closely related taxa that are genetically and morphologically (male horn shape) divergent from each other. *Dynastes ecuatorianus* and *Dynastes lichyi* (Cloud forest of the eastern slope of the Northern Andes, that is, Colombia, Ecuador, and Peru) are genetically and morphologically distinct non‐sister taxa that can be found in geographic proximity (e.g., in the Napo province of Ecuador), which implies the establishment of reproductive isolation

There are natural circumstances where speciation can be achieved without population subdivision, and where population subdivision may not lead to complete speciation. For example, speciation can be completed without population subdivision in cases of polyploidy evolution (Van de Peer, Mizrachi, & Marchal, 2017; Wood et al., 2009). On the other hand, although geographic isolation and ecological specialization often result in genetically structured populations, these populations may not persist long enough to become distinct species or divergence may not be strong enough to maintain a stable species boundary (Baker, 2005; Dynesius & Jansson, 2014; Harvey, Singhal, & Rabosky, 2019; Nosil, Feder, Flaxman, & Gompert, 2017; Nosil, Harmon, & Seehausen, 2009; Sterner, 2017). Empirically, recent studies have shown that the level of intraspecific genetic divergence does not predict speciation rate, indicating that structured populations often fail to become species (Huang & Knowles, 2016; Singhal et al., 2018). Nevertheless, many recent studies refer to the continuous process of biological divergence as a “speciation continuum,” implying that population subdivision (view 2; Figure 1) is only an early stage of speciation (view 1; Figure 1).

The Darwinian view of speciation implies that population subdivision is speciation, because Darwin drew no lines between varieties and species (page 147 in Mayr, 1942; but see Mallet, 2008). The concept of biological species introduced during the modern synthesis redefined speciation emphasizing the importance of reproductive isolation (Baker, 2005; Mayr, 1942; Wilson & Brown, 1953) (Figure 1). Specifically, population subdivision was defined as the first phase of speciation where diverging taxa were formed. However, speciation may fail to complete if reproductive isolation, the second phase of speciation, had not evolved upon secondary contact (Mayr, 1942). Reproductive isolation can arise as a byproduct of the divergence process or as an adaptive response to secondary contact (Baker, 2005). Currently, speciation is often used as an umbrella term that encompasses all processes related to the origin and maintenance of biological divergences between taxa, and the evolution of reproductive isolation can be viewed as a tipping point (Nosil et al., 2017) that the diverging taxa have evolved special features to prevent them from merging into a single evolutionary lineage. Unlike the constant debates about how to define and determine biological species (De Queiroz, 2007; Sterner, 2017), experts unhesitantly refer to processes responsible for both intraspecific and interspecific divergences as speciation.

## 2 RAMIFICATIONS OF THE POPULATION SUBDIVISION VERSUS SPECIATION CONUNDRUM

The use of “speciation” to encompass many different processes that may be responsible for the origin versus maintenance of biological divergences can have profound consequences. For example, the assumption that a period of complete spatial isolation is required for completing animal speciation has recently been challenged by many empirical examples (i.e., speciation with gene flow; Nosil, 2008). However, were the geographic taxa from the empirical studies actually representing different “good species”? Geographic isolation (either by distance or physical barriers) is often associated with local phenotypic forms in animals (races, forms, or subspecies that represent the incipient stage of speciation; Mayr, 1942). Genetic interchange between geographic forms would not be an unexpected pattern even in strict allopatric speciation, because the divergence between geographic forms, or incipient species, only represents the first phase of speciation. Many incipient species may fail to become distinct species (e.g., there are a lot more subspecies than species in birds; see pages 155–156 in Mayr, 1942), and high frequency of gene flow between diverging taxa can be one reason why allopatric speciation often fails to complete. That is, strict allopatric speciation rejected by recent empirical studies may actually support allopatric speciation outlined by Mayr (1942), because many of the empirical studies used cases that would only be categorized as geographic forms instead of species based on conventional taxonomic practices. These geographic forms may either be evolutionarily transcendent or ephemeral (Dynesius & Jansson, 2014; Sterner, 2017), but reproductive isolation has certainly not yet been fully established. To reject allopatric speciation, we need to determine whether gene flow has occurred in the second phase of allopatric speciation, the evolution of reproductive isolation, between species where incompatibility has been fully developed (i.e., between strict biological species).

**FIGURE 2:**
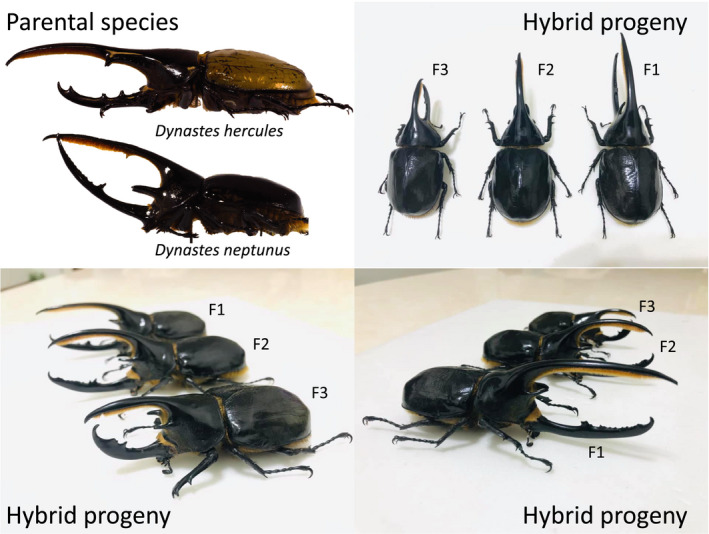
Exemplar hybrid progeny between *Dynastes hercules* and *Dynastes neptunus*. The images of the hybrid individuals and progenies were produced by Mr. Zhiyong Lin

The assumption that incompatibility will arise and can be used as defining criterion for biological species, however, is equally controversial. The significance of evolving incompatibility between species has been extensively discussed (Baker, 2005; Powell et al., 2020), but there is also support for why incompatibility may not evolve between closely related diverging taxa, which in my opinion has received less attention. Firstly, incompatibility can be a byproduct of the divergence process. Because it is a byproduct of divergence, however, the evolutionary time needed for incompatibility to evolve cannot be predicted. For example, fertile and viable offspring in the Hercules beetle system can be produced between species of different subgenera, representing >11 million years of divergence (the generation time of Hercules beetles ranges from 2 to 3 years; see Huang & Knowles, 2016 for estimates of divergence times; Figure 2). The hybrid progeny lasted at least three generations (Figure 2). Why would we expect incompatibility between closely related species if incompatibility could not have fully developed between even distantly related species? Secondly, the production of infertile or inviable offspring is disastrous for the evolutionary fitness of individuals (Baker, 2005), but empirical studies have found that closely related geographic taxa often hybridize in parapatry (Abbott et al., 2013; Nosil, 2008). Why would such a harmful evolutionary development, that is, incompatibility, be favored that greatly reduces the fitness of individuals? Thirdly, the evolution of incompatibility at the species level will only limit the source of genetic variation for a species to cope with future environmental changes. Because the Earth environment has never been static, with climatic and/or geological conditions constantly changing, species that do not evolve incompatibility and can exchange genetic material with other species would have an advantage and be selectively favored in an ever‐changing world (Hamilron & Miller, 2016). Note that incompatibility evolves between fully diverged biological species in nature (e.g., Powell et al., 2020), but the empirical examples demonstrating the evolution of incompatibility are often not between sister species, or diverging taxa (Kang, Schartl, Walter, & Meyer, 2013). The filtering stage that delineates intraspecific from interspecific variations, that is, the evolution of incompatibility, strongly favored by some conventional taxonomists, may not be an appropriate criterion distinguishing intraspecific from interspecific divergences.

In the following sections, I will briefly review the intertwining history of speciation and species delimitation studies where the divergence process is somewhat continuous, mainly from a geographic perspective. The spatial variation of biodiversity patterns has been a focus of study from the beginning of evolutionary biology (e.g., Charles Darwin and the Galapagos Finches) to the modern synthesis (e.g., Ernst Mayr and the birds of Papua New Guinea) to today. The pattern is interesting because the mechanisms that have generated and maintained spatial variation pertain to the origin, maintenance, and accumulation of biodiversity. I will briefly outline what phylogeographic studies have revealed about the process of evolutionary divergence, that is, population subdivision and/or speciation, regardless of what anyone may call it. Objective and consistent species delimitation once seemed possible during the development of the field, which could have helped settle the population subdivision versus speciation conundrum. However, current knowledge necessitates discussing confounding effects between species delimitation and speciation studies. It is also necessary to consider the continuing challenges of interpreting results from studies that delineate not only intraspecific versus interspecific biological patterns, but also processes.

## 3 FROM PHYLOGEOGRAPHY TO SPECIES DELIMITATION

The field of phylogeography began with the development of DNA sequencing, which allowed for statistical examination of genetic variation and phylogenetic relationships across closely related geographic taxa (Avise, 2000; Templeton, 1998). Many evolutionary models, statistical methods, and computer programs have been developed to test if and how genetic divergence has occurred spatially in different biological systems (Knowles, 2009). Specifically, approaches that incorporate population genetics have become a focus in phylogeography. Several advances even changed our long‐held perceptions of how speciation proceeds, for example, the theory of porous genomes and genomic islands of speciation (Wu, 2001), the isolation with migration model (Hey & Nielsen, 2004), and the rejection of strict allopatric speciation in many empirical systems (Nosil, 2008). The advances in the field—theories, models, analytical methods, and data interpretations—were not without constant challenges (e.g., Cruickshank & Hahn, 2014; Knowles, 2008), but our understanding of how genetic divergence can be spatially structured and maintained has undeniably greatly improved during the approximately two decades of phylogeographic and speciation studies (Avise, 2009; Knowles, 2009). The aim of phylogeography is to understand the history and formation of “species,” but one question that phylogeographic studies might also try to answer is whether the different spatial genetic entities merit species status. The subsequent taxonomic revision based on what was learned from phylogeographic studies was sometimes practiced based on a phylogenetic species concept (Carstens, Pelletier, Reid, & Satler, 2013). Although there had been an increase to assess whether there were phylogenetic species in their systems after the development of DNA barcoding (Hebert, Cywinska, Ball, & DeWaard, 2003), the use of taxonomic ranks—populations, subspecies, or species—of the systems in phylogeographic studies were mostly based on conventional taxonomic studies, even though they have been inconsistent across biological systems (Hey & Pinho, 2012). As a result, the processes leading to the divergence patterns among different empirical systems, although they might all have been referred to as speciation, may have involved many different mechanisms.

The inclination of phylogeographers to amend taxonomies changed after a seminal study of genetic divergence across geographic taxa in a group of phenotypically and ecologically similar geckos in West African rainforests (Leaché & Fujita, 2010). The authors used the Bayesian Phylogenetics and Phylogeography program (Yang & Rannala, 2010), which implements the multispecies coalescent model (Degnan & Rosenberg, 2009) to delineate independently evolving lineages (or panmictic genetic clusters; Sukumaran & Knowles, 2017). Although some conventional taxonomists objected the new coalescent‐based taxonomic treatments (Bauer et al., 2011), a subsequent study formally described the statistically delimited gecko phylogeographic units as species (Wagner, Leaché, & Fujita, 2014) by applying the general lineage concept, defining species as genetically connected metapopulations that form independently evolving lineages (De Queiroz, 2007). Statistical and integrative model‐based species delimitation has become a research focus in systematics, encouraged by the success and promise that consistent taxonomic treatments could have in a model‐based framework (Fujita, Leaché, Burbrink, McGuire, & Moritz, 2012) and the need to officially describe species to affect studies of biodiversity (Carstens et al., 2013). Many empirical systems that were once studied phylogeographically were reinvestigated for species delimitation with revised taxonomic treatments (c.f., Chambers & Hillis, 2020; Pyron & Burbrink, 2009; Ruane, Bryson, Pyron, & Burbrink, 2014).

## 4 SPECIES DELIMITATION AND SPECIATION

Studies of phylogeography and species delimitation often use the same research methodology that focuses on the same natural pattern—the population structure of genetic divergence—and use the same data sets—molecular loci and/or phenotypic data—and geographic sampling design and apply the same analytical models and approaches, for example, coalescent model and approximate Bayesian computation. Studies of species delimitation were thus successors of phylogeographic studies that used more data and refined models (Freudenstein, Broe, Folk, & Sinn, 2017). The scientific outputs of studies of phylogeography and species delimitation, however, have one main difference. Phylogeographic studies aim to understand the historical process that generate and structure genetic divergence among geographic taxa (populations, subspecies, or species; Avise, 2000), and studies of species delimitation further emphasize determining the number of genetic units that merit species status. Taxonomic revisions or descriptions of new species are then often expected.

Do we really have a better understanding of what species are, or the best methodology for delineating species boundaries from phylogeographic and species delimitation studies? Specifically, the field has transformed from studying evolutionary history among taxa, where the taxonomic rank has often been predetermined by other experts, to redetermining the taxonomic rank of the taxa after studying their evolutionary histories and the level of genetic divergence. Both genetically structured populations and species are evolutionary lineages (as are all intermediate taxonomic ranks such as subspecies and ecotypes), and enough data could provide the statistical power to differentiate such lineages (Sukumaran & Knowles, 2017; Huang 2018). If we do not understand how intraspecific and interspecific divergences were generated and maintained in a study system, the addition of more data will only lead to the splitting of finer scale genetic divergences into genealogical species; the addition of more data and models may not help with objectively determining species boundaries that are biologically and evolutionarily relevant. Conventional taxonomic practices have often been criticized, where different taxonomic decisions are made using different data sets (Avise & Liu, 2011; Hebert et al., 2003). Similarly, different species delimitations could be produced when different molecular/phenotypic data sets are used (Pyron, Hsieh, Lemmon, Lemmon, & Hendry, 2016; Huang 2018). That is, the taxonomic inferences from studies of coalescent‐based molecular species delimitation can be as artificial as those from studies using conventional taxonomic practices that are strongly determined by the choices of data sets (characters), analytical methods, and sampling designs (e.g., Jackson, Carstens, Morales, & O'Meara, 2017). Through a thorough understanding of the divergence processes, instead of overemphasizing the statistical power generated by the ever‐increasing available data sets and the efficacy of the models to detect genetic divergences, we may be able to make evolutionarily coherent decisions about species boundary. A thorough understanding of the divergence processes however may also depend on the available data sets and the efficacy of the models to discriminate among possible historical processes, which make the speciation and species delimitation studies even more complicated.

## 5 CONCLUSION AND FUTURE PERSPECTIVE—STUDYING THE CONTINUITY/DISCONTINUITY OF DIVERGENCE

Is population subdivision speciation? The question has not been as contentious as “what is a species?” in evolutionary biology. However, the answer to the question is equally important and may have impacts on species definition. The aim of this study is not to burden evolutionary ecologists with semantics (e.g., population subdivision vs. speciation; Figure 1). The term speciation encompasses many different processes, and the focus of the study is to emphasize that some, if not many, of the processes generate biological divergences that may never translate into species diversity. The consequence of the imprecise, or inconsistent, use of “speciation” can result in different evolutionary inferences made between studies simply because “your speciation is not what I mean by speciation” even when both studies investigated the same type of biological divergence (e.g., allopatric mode of speciation). Additionally, our ability to objectively delineate evolutionarily relevant biological species can be compromised. Before quoting the term speciation or speciation continuum, we may need to ask ourselves do I study the divergence history between two geographic populations (or host‐plant races), between two subspecies (or eco‐forms), or between two reproductively isolated species? Furthermore, does population subdivision equate to incomplete speciation (c.f., Nosil et al., 2009)?

I argue that identifying the sources that cause the contention could be empirically helpful, although advancing beyond the philosophical contention may be difficult. Studying the variation in the continuity of divergence among biological systems may be a good start to identify the source of this philosophical contention. Divergence can be continuous in the case of ring species, where genetic/phenotypic divergence has different levels, implying different levels of reproductive isolation, when comparing different pairs of geographic taxa (Moritz, Schneider, & Wake, 1992). The process can be genetically continuous, where divergent genomic islands gradually accumulate and expand through time. Divergence can also be completed instantaneously in the formation of polyploids, where incompatibility is spontaneous. The continuity of divergence, however, would probably be somewhere between the extremes in most biological systems. The ability or inability to identify natural breaks along a continuous process could be associated with different views on whether population subdivision is or is not speciation.

Investigating evolutionary divergences in multiple biological systems is challenging due to the distinct biological, ecological, and genetic properties that characterize different biological systems, but these properties are key to understanding the mechanisms and conditions that generate, structure, and maintain patterns of biodiversity. Specifically, understanding the origin of intraspecific variation, the structured intraspecific variation that subsequently leads to interspecific divergence, and the maintenance of interspecific diversity in different biological systems may ultimately help us answer central questions in evolutionary biology: (1) how have so many species evolved, (2) what are species, (3) are species natural or artificial entities, and (4) do the answers to questions 1–3 depend on the biological system?

## CONFLICT OF INTEREST

There is no competing interest.

## AUTHOR CONTRIBUTION

**Jen‐Pan Huang:** Conceptualization (equal); funding acquisition (equal); investigation (equal); resources (equal); validation (equal); visualization (equal); writing – original draft (equal); writing – review and editing (equal).
